# Supplementary material for: Evidence supported by Mendelian randomization: impact on inflammatory factors in knee osteoarthritis
Source: Front Med (Lausanne). 2024 May 28;11:1382836. doi: 10.3389/fmed.2024.1382836 (PMC11165061; doi:10.3389/fmed.2024.1382836)
Supplement: Supplementary file 1 [file Data_Sheet_1.ZIP › data/GCST90274805_5e6/805.forest.pdf]

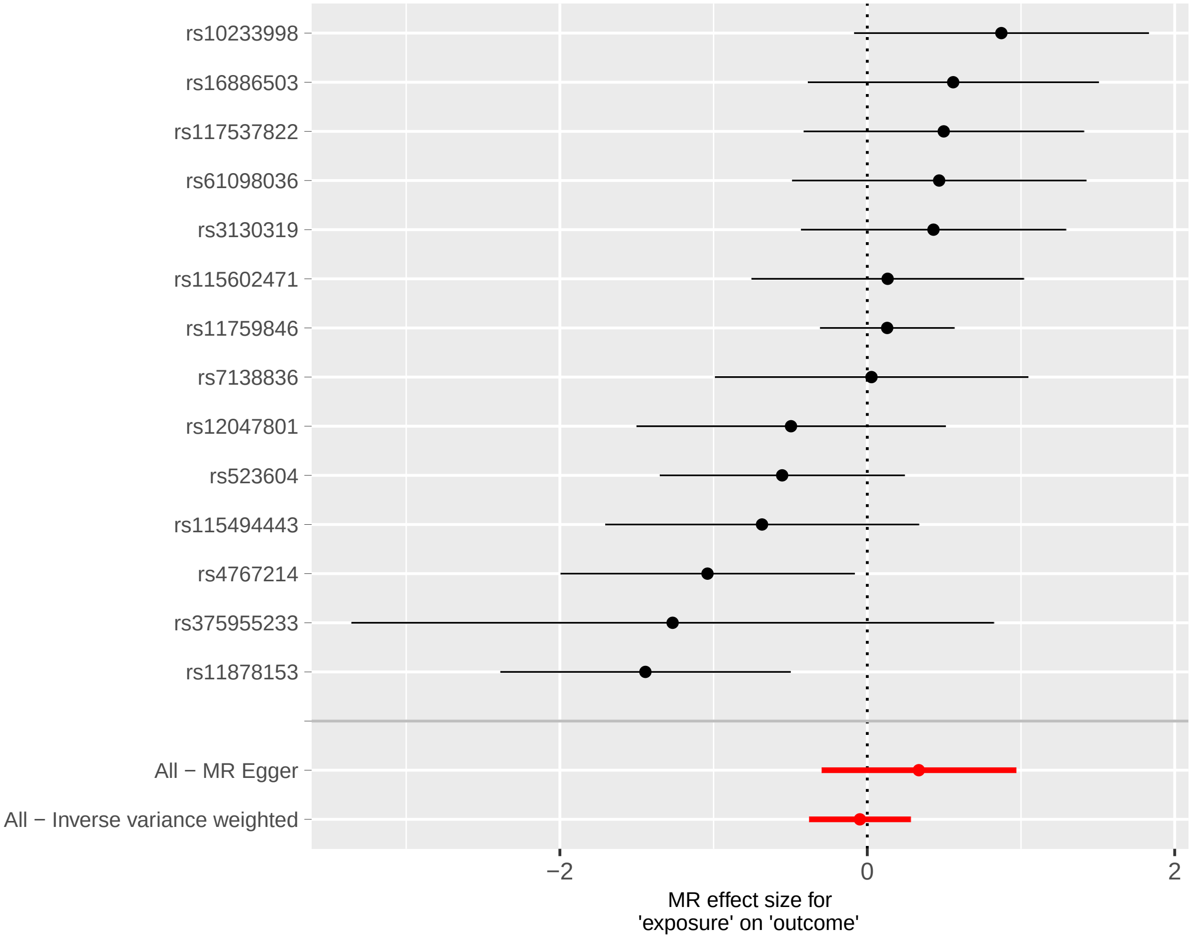
rs10233998
rs16886503
rs117537822
rs61098036
rs3130319
rs115602471
rs11759846
rs7138836
rs12047801
rs523604
rs115494443
rs4767214
rs375955233
rs11878153
All − MR Egger
All − Inverse variance weighted
−2
0
2
MR effect size for
'exposure' on 'outcome'
